# Supplementary material for: The 2023 Mw 7.8–7.7 Kahramanmaraş earthquakes were loosely slip-predictable
Source: Commun Earth Environ. 2025 Feb 5;6(1):80. doi: 10.1038/s43247-024-01969-5 (PMC11798867; doi:10.1038/s43247-024-01969-5)
Supplement: Supplementary file 2 — Supplementary information [file 43247_2024_1969_MOESM2_ESM.pdf]

Supplementary Information for

The 2023 M_w 7.8-7.7 Kahramanmaraş earthquakes were loosely slip-predictable

Ellis Vavra[1], Yuri Fialko[1], Fatih Bulut[2], Aslı Garagon[2], Sefa Yalvaç[3], Cenk Yaltırak[4]

[1]University of California, San Diego, Scripps Institution of Oceanography, 92093, California, USA
[2]Boğaziçi University, Kandilli Observatory and Earthquake Research Institute, Geodesy Department 34684, Istanbul, Turkey
[3]Gümüşhane University, Faculty of Engineering, Survey Engineering Department, 29000, Gümüşhane, Turkey
[4]Istanbul Technical University, Faculty of Mines, Geology Department, 34467, Istanbul, Turkey

Supplementary Notes

Aftershocks

The seismic network operated by Boğaziçi University, Kandilli Observatory and Earthquake Research Institute detected more than 16,000 aftershocks within the first 40 days following the mainshocks[1]. The aftershock magnitudes range from M 0.8 to 6.6. This extensive aftershock data allows determining the extent and geometry of the rupture planes associated with the mainshocks. In particular, M3+ aftershocks provide sufficient epicentral accuracy to locate the rupture planes associated with the February 6, 2023, earthquakes.

Locking depth comparisons

We perform two independent estimates for the locking depth of each major fault segment that slipped in the 2023 Kahramanmaraş doublet, using 2D inversions of GNSS data and the depth extent of seismicity. We compare these to depth-averaged profiles of coseismic slip (Fig. S1). For each fault segment, we estimate the average slip at 1 km depth intervals by extracting the corresponding slip values from each coseismic slip model and performing a weighted average, with patch lengths as the weighting factors.

Supplementary Discussion

Alternate rupture scenarios

In addition to the preferred earthquake history based on the integration of paleoseismic evidence and epicenters and rupture extents from historical records, as presented in the main text, we consider several additional scenarios to account for uncertainties in the published historic earthquake data.

The felt area and epicenter of the 1893 M 7.1 earthquake are relatively well-constrained to have occurred on the Erkenek segment of the EAF[2], so we do not consider any alternative scenarios. While we do not consider alternative scenarios along the

northern EAF, we note that a magnitude ~6.8 event may have occurred on the Çardak segment in 1544[3]. Thus, the accumulated slip deficit since the 584 earthquake is an upper bound, indicating that the northern EAF segments may be in better agreement with the slip-predictable model than would be inferred from considering the 584 event alone (Fig. 5).

Paleoseismic evidence confirms the occurrence of the 1513 M 7.4 event on the southern portion of the Pazarcık segment, but not near its northern margin at the Erkenek segment[4]. If the 1513 rupture terminated shortly beyond the Tevekkelli trench site, located ~20 km northeast of the southern end of the Pazarcık segment, then a significant portion of the Amanos segment would have likely also failed in order to generate the estimated magnitude of 7.4[5]. We consider this scenario in Figure SXX, in which case we observe that the Amanos segment is loosely slip-predictable.

In the event that none of the 1513, 1822, or 1872 events ruptured the Amanos segment, then the most recent major earthquake has been previously suggested to have occurred in 521[6,7] or 601[8,9]. While the 1114 M ≥ 7.8 earthquake has not typically been considered to have ruptured the Amanos segment, its confirmation along the adjacent Pazarcık segment through paleoseismology and the large inferred magnitude suggest that a significant portion of the Amanos or Erkenek (or both) would have also slipped during this event. Similarly, if the Amanos segment did not participate in the 1513 rupture, then a significant portion of the Pazarcık segment is required to have slipped to generate the inferred magnitude of 7.4.

To summarize, a lack of rupture along the central-to-northern Pazarcık in 1513 suggests that instead the Amanos segment ruptured. Conversely, if the Amanos segment did not rupture in 1513, then the Pazarcık segment nearly fully ruptured. Thus, it is required that either the Amanos or Pazarcık segment slipped in 1513 and the 1114 M ≥ 7.8 can be considered a lower bound for only one event, not both. This logic does not preclude the concurrent rupture of both the Amanos and Pazarcık segments in 1513; however, we are primarily concerned in constraining the oldest possible preceding events, and this scenario is less of interest from the perspective of analyzing pre-2023 slip deficits. In any scenario where the 1114 earthquake was the preceding major event on the Amanos or Pazarcık, the corresponding increase in the accumulated slip deficit suggests that interseismic strain equivalent ~2 m of slip is still harbored by either segment, at minimum.

In addition, within the last decade a previously unnoted M > 7 event in 1795 was suggested to have occurred in the vicinity of Kahramanmaraş , with plausible host faults being the Kahramanmaraş fault, the Engizek fault, and the Pazarcık or the Çardak segment of the EAF[9,10]. The lack of evidence in paleoseismic trenches seems to rule out major rupture along the Çardak segment[3], but we consider the scenario in which the 1795 event was the last major earthquake on the Pazarcık segment.

Impact of time-dependent slip rates on slip deficit estimates

It is likely that geodetic slip rate estimates represent an upper-bound, and the geologic rates represent a lower-bound, on the long-term fault slip rates. In the view that the slip rate discrepancy is a result of observational sensitivity to more complex accommodation of a constant strain rate, then averaging over all available rate estimates is reasonable. However, it cannot be ruled out that the slip rate on the EAF may not have been stationary over millennial time scales[11,12]. The influence on variable fault slip rates on the accumulated slip deficits depends on the character and timing of observed acceleration. Studies that have inferred fault acceleration and deceleration broadly constrain the duration of rate-changes from several hundred years to two millennia, but are limited in resolving their detailed time-dependent character[11]. If we assume that hypothetical acceleration along the EAF occurred linearly from some point in the past up until February 2023, we can estimate the acceleration duration that would produce a slip deficit equivalent to that associated with a constant slip rate (i.e. estimates in Fig. 5). If we take the constant slip rate to be the average of all slip rate estimates v_{avg} and assume the fault accelerated from the geologic slip rate $v_{geologic}$ to geodetic slip rate $v_{geodetic}$ then given the interval since the last earthquake Δt_{eq} we can calculate the time period Δt_{acc} to produce an equivalent slip deficit,

$$\Delta t_{acc} = \frac{2\, v_{avg}}{v_{geologic} + v_{geodetic}} \Delta t_{eq} \ .$$

Performing this calculation, we find that acceleration intervals Δt_{acc} range from Δt_{eq} to 1.1 Δt_{eq}. So, if acceleration from the geologic slip rates to geodetic slip rates occurred roughly over the interseismic interval preceding the 2023 doublet, it would not significantly affect our conclusions. Given these intervals range from 130-1439 years for the EAF, this would be compatible with previously inferred acceleration/deceleration periods along other faults[11]. If acceleration occurred over much shorter or longer time periods, we would observe a more significant change in our estimated slip deficits. More prolonged acceleration would increase the slip deficit, as slip would be accumulating at rates closer to the fast geodetic slip rates over the preceding interseismic interval, and vice versa for acceleration concentrated toward the end of the interseismic period. Occurrence of damaging earthquakes during ~500-1100 CE and a subsequent relative quiescence between ~1100-1700 CE could be interpreted as periods of accelerated and decelerated slip rate, respectively[13], but would reflect an opposite pattern to the observed high geodetic slip rates and low geologic slip rates. Along at least the Pazarcık segment, geological evidence suggests the fault slip rate has remained relatively constant over a period of 18 kyr[4]. In general, century-long geodetic observations world-wide do not lend support to variations in the interseismic loading rates[4,5,38].

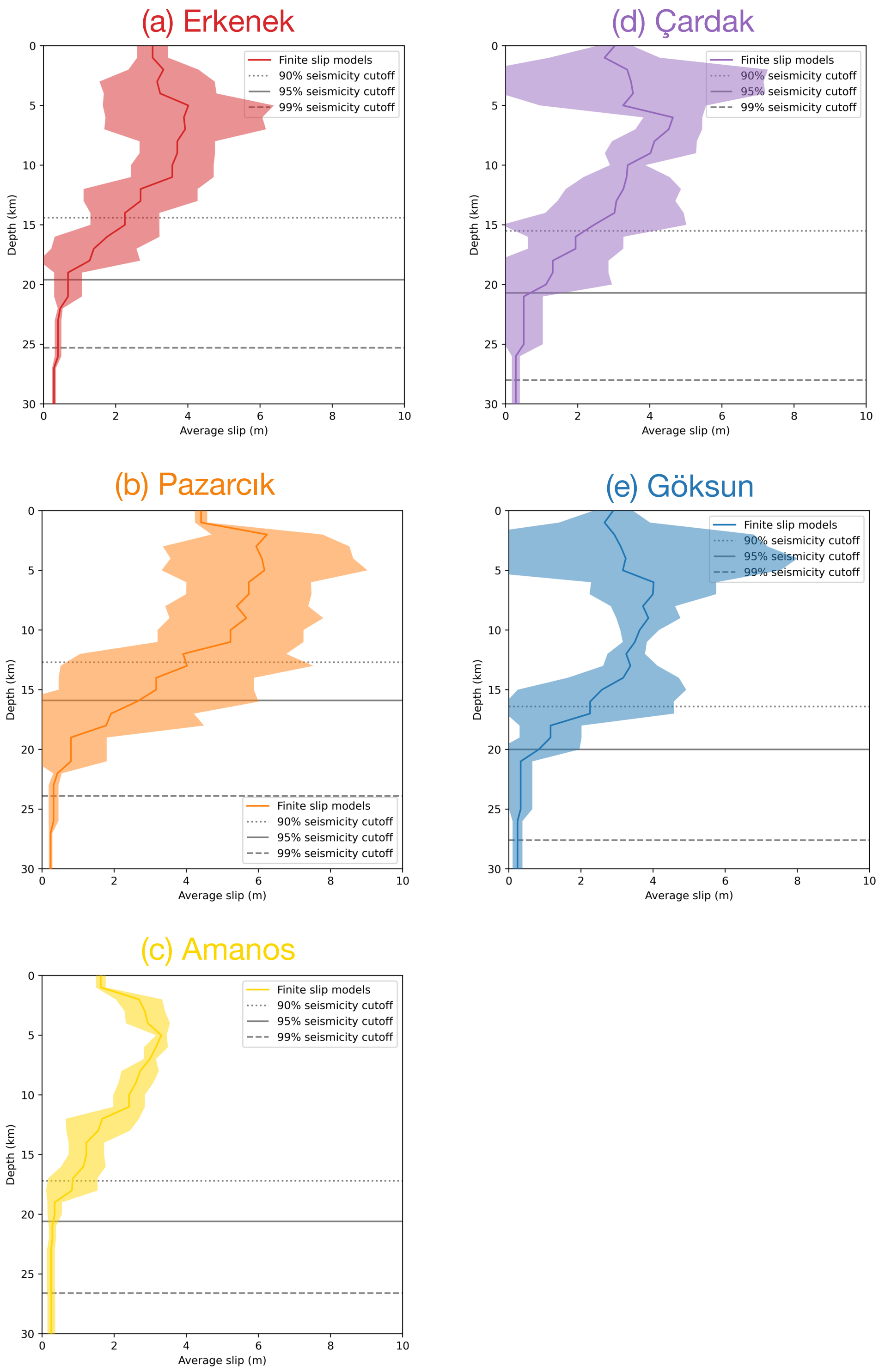

Supplementary Fig. 1 | Comparison of locking depth estimates from the KOERI earthquake catalog with depth-averaged coseismic slip for the (a) Erkenek, (b) , (c) Amanos, (d) Çardak, and (e) Göksun segments of the EAF. The curve and associated

shading indicate the mean and standard deviation of slip extracted from the cosesimic finite slip models.

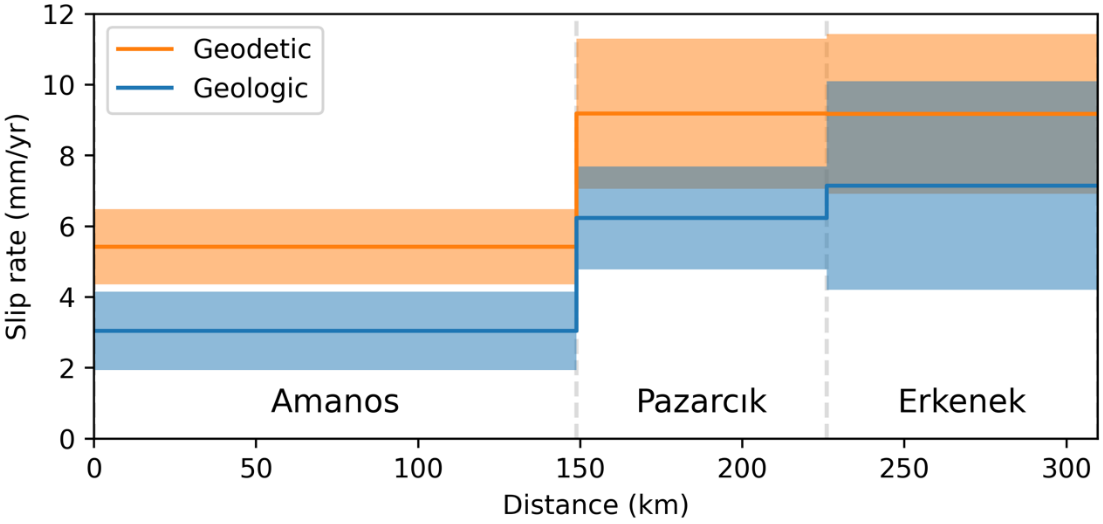

Supplementary Fig. 2 | Comparison of slip rates along the main EAF obtained from geologic and geodetic methods. In general, modern geodetic slip rates are 2-3 mm/yr larger than those estimated from long-term fault offsets (1000s of years). Individual geologic and geodetic slip rates are listed in Table S2.

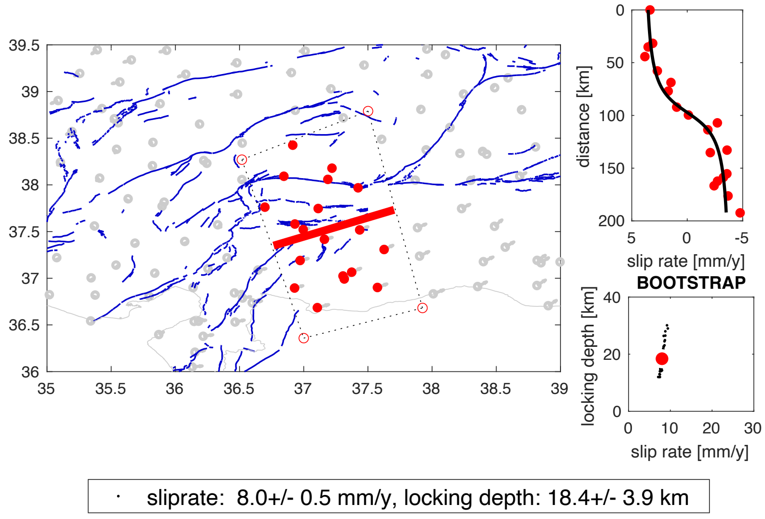

Supplementary Fig. 3 | Results from the dislocation modeling of interseismic GPS slip rates across the Pazarcık segment of the EAF.

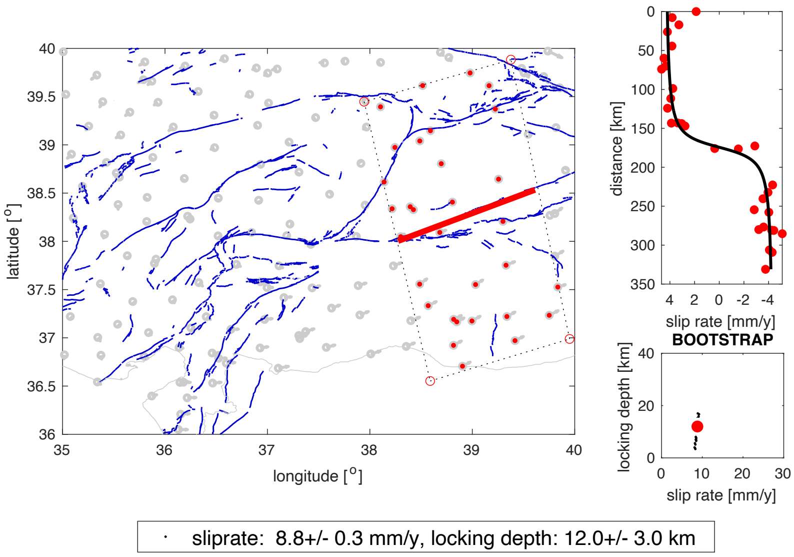

Supplementary Fig. 4. | Results from the dislocation modeling of interseismic GPS slip rates across the Erkenek segment.

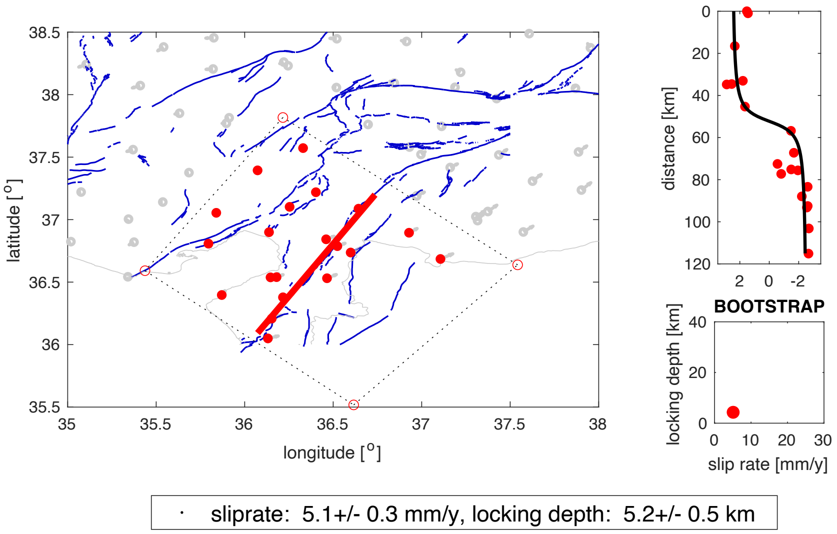

Supplementary Fig. 5 | Results from the dislocation modeling of interseismic GPS slip rates across the Amanos segment.

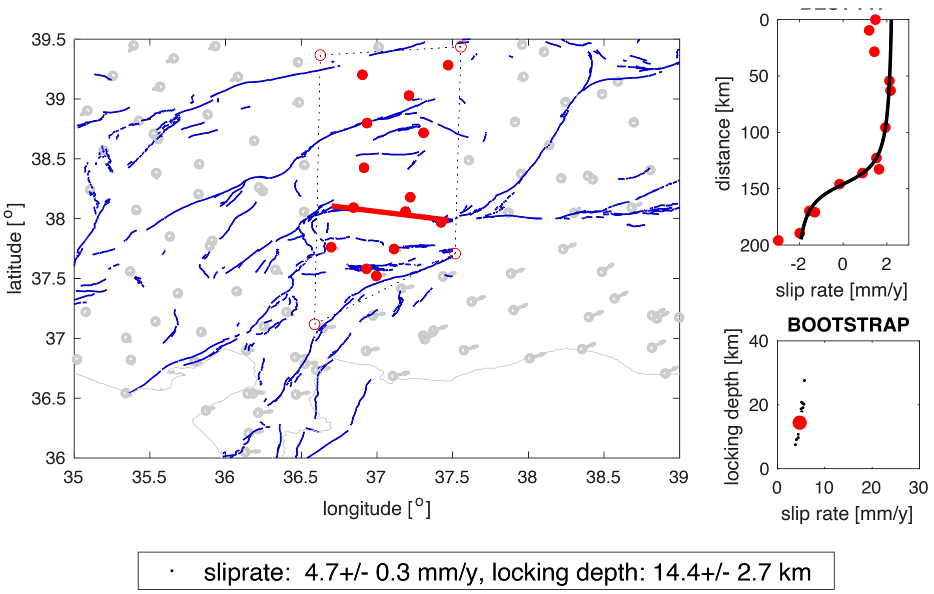

Supplementary Fig. 6 | Results from the dislocation modeling of interseismic GPS slip rates across the Çardak Fault.

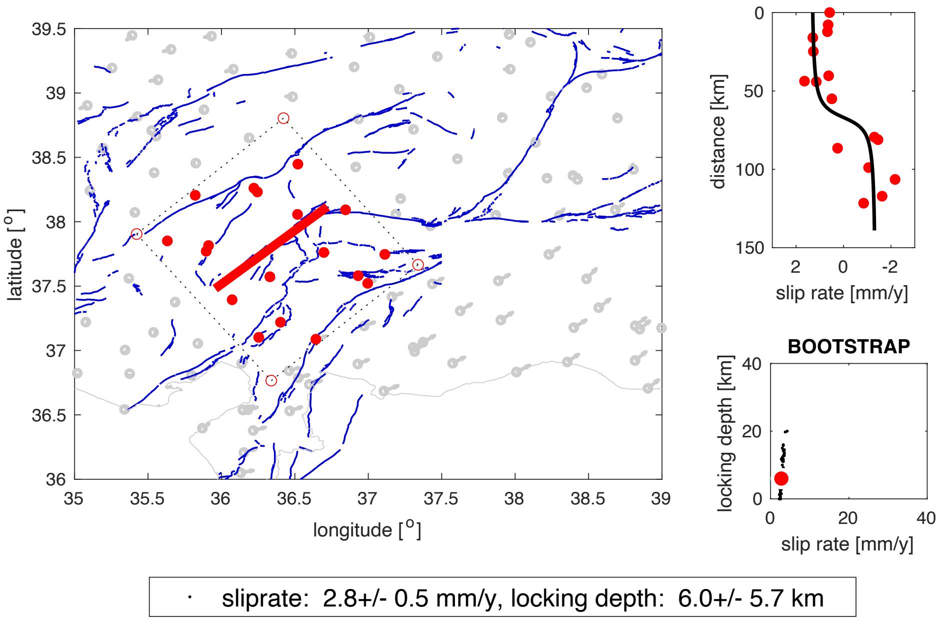

Supplementary Fig. 7 | Results from the dislocation modeling of interseismic GPS slip rates across the Göksun Fault.

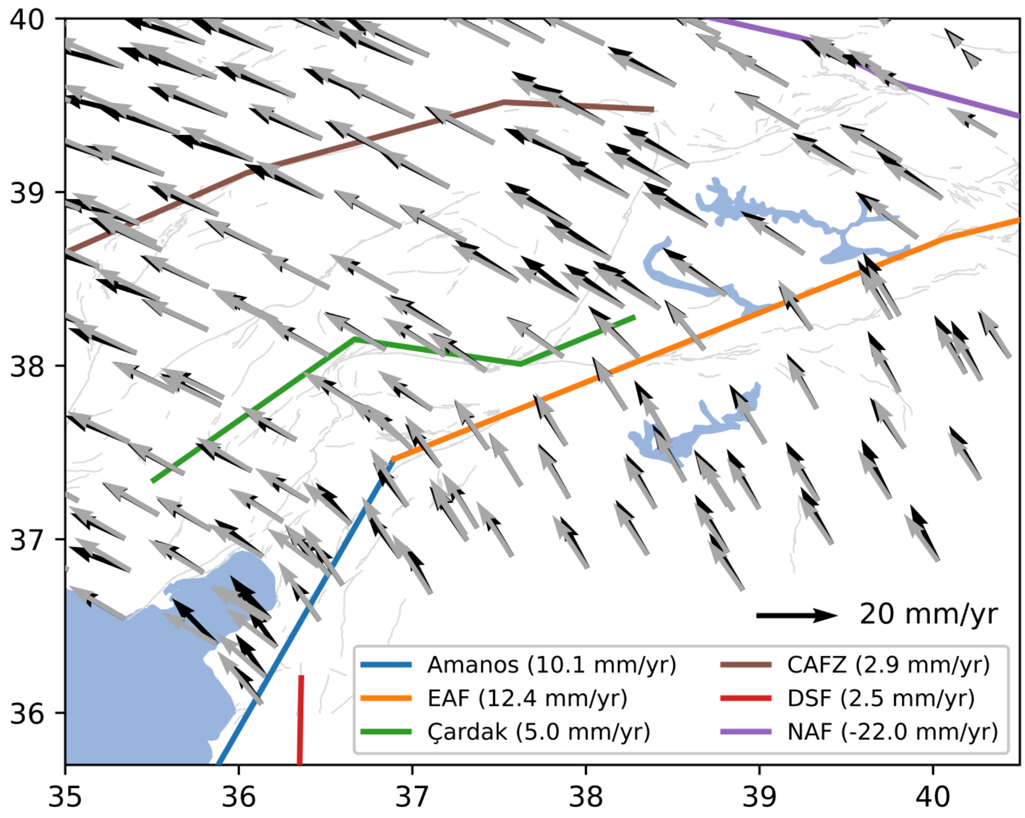

Supplementary Fig. 8 | Observed horizontal GPS velocities (black arrows) and predictions of a best-fit 3-D fault model (gray arrows). Color lines denote modeled faults. The inferred slip rates are indicated in the legend. Slip rates on the Dead Sea Fault (DSF) and North Anatolian Fault (NAF) are prescribed. Thin gray lines denote other geologically mapped faults.

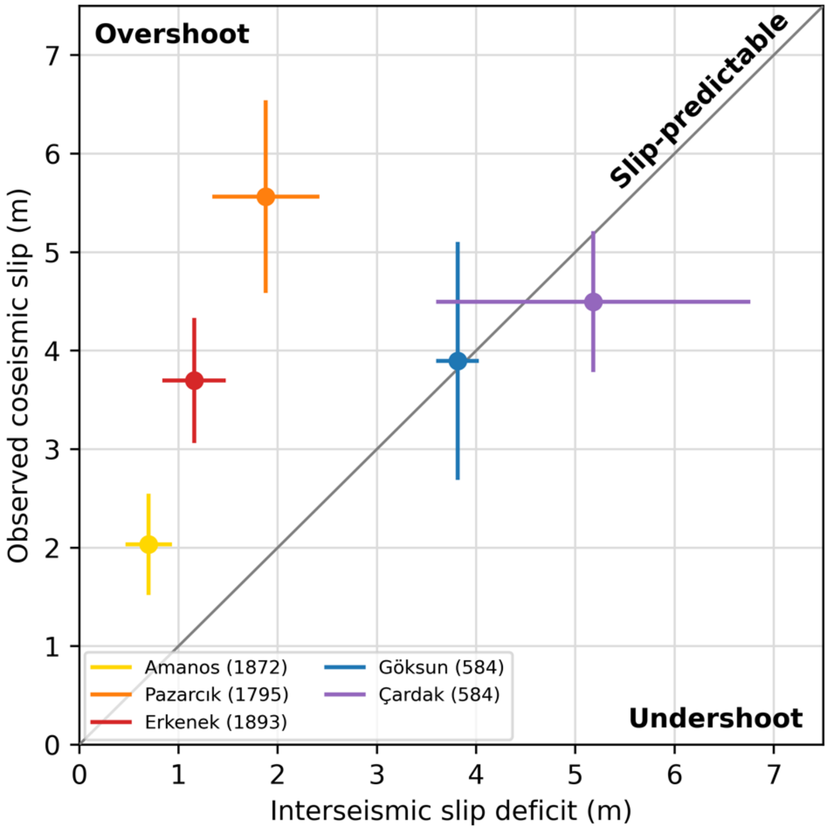

Supplementary Fig. 9 | Same as Fig. 5, but for the case where the Pazarcık segment last ruptured in 1795.

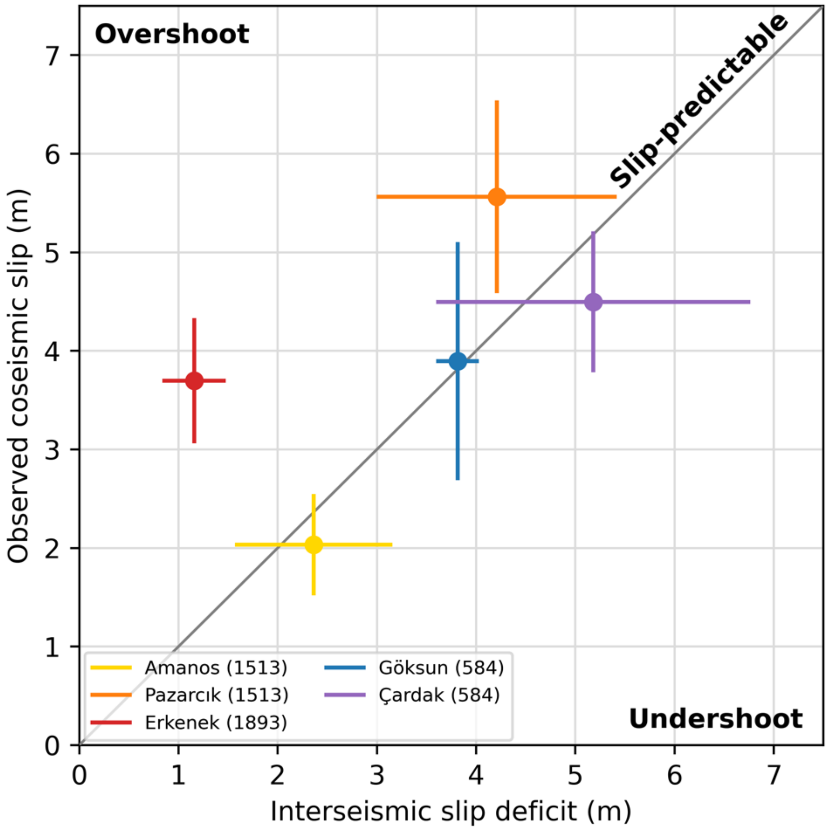

Supplementary Fig. 10 | Same as Fig. 5, but for the case where both the Amanos and Pazarcık segments last ruptured in 1513.

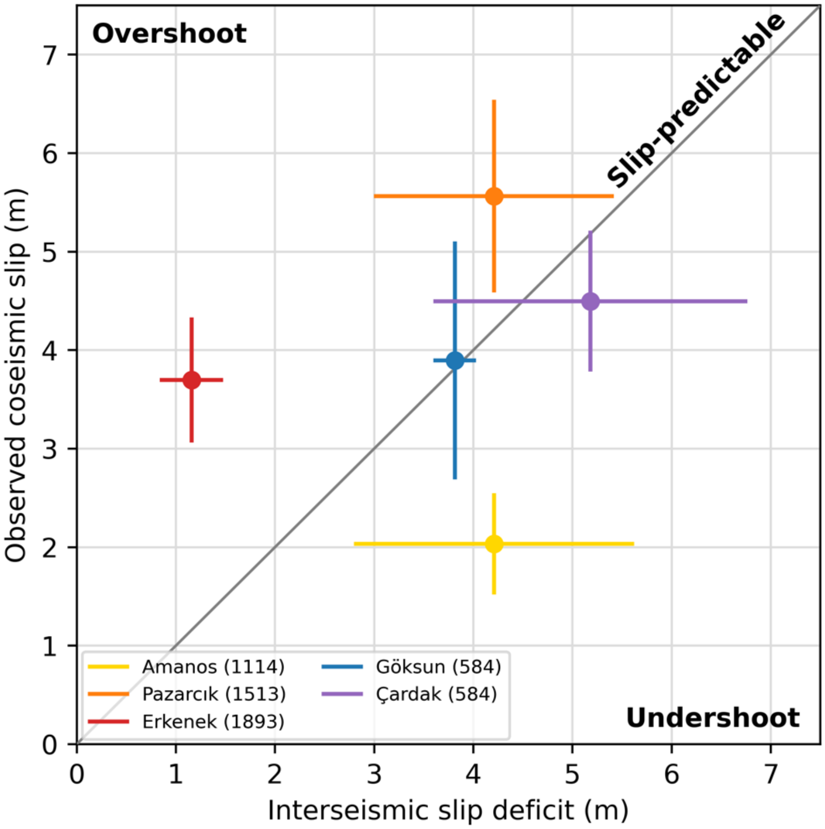

Supplementary Fig. 11 | Same as Fig. 5, but for the case where the Amanos segment last ruptured in 1114.

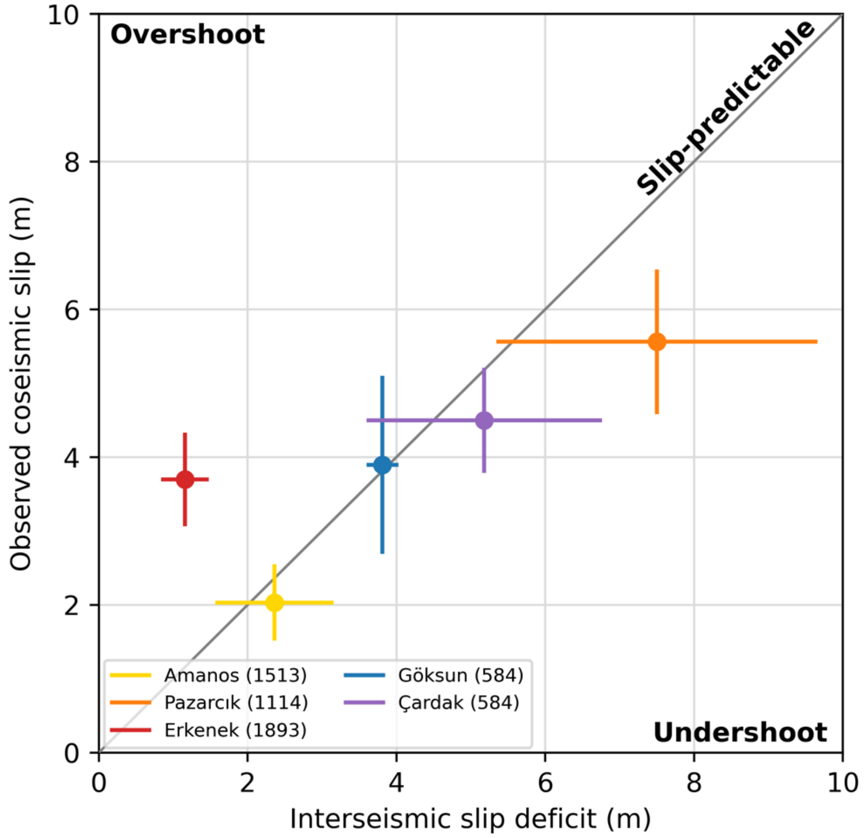

Supplementary Fig. 12 | Same as Fig. 5, but for the case where the Amanos segment last ruptured in 1513 and the Pazarcık segment in 1114.

Table S1 | Cosesimic slip models used in this study.

**Reference**	**Input Datasets**	**Medium**
Barbot et al. (2023)[14]	Static GNSS, InSAR	Homogeneous
Goldberg et al. (2023)[15]	Seismic, high rate GNSS, static GNSS, InSAR	Layered
Jia et al. (2023)[16]	Static GNSS, InSAR	Homegenous
Jia et al. (2023)[16]	Static GNSS, InSAR	Layered
Ren et al. (2024)[9]	Seismic, high rate GNSS, static GNSS, InSAR	Layered

Table S2 | Fault slip rate estimates used in this study.

Study	Segment	Slip rate (mm/yr)	Error (mm/yr)	Type	Method
This study	Amanos	5.1	0.3	Geodetic	2D dislocation
This study	Cardak	4.7	0.3	Geodetic	2D dislocation
This study	Erkenek	8.8	0.3	Geodetic	2D dislocation
This study	Goksun	2.8	0.5	Geodetic	2D dislocation
This study	Pazarcık	8	0.5	Geodetic	2D dislocation
Yönlü & Karabacak (2024)[4]	Pazarcık	5.6	0.3	Geologic	Offsets
Li et al. (2023)[17]	Erkenek	4.5	0.16	Geodetic	2D dislocation
Li et al. (2023)[17]	Pazarcık	5.5	0.31	Geodetic	2D dislocation
Li et al. (2023)[17]	Amanos	3.5	0.35	Geodetic	2D dislocation
Özkan et al. (2023)[18]	Amanos	5.49	1.12	Geodetic	Block model
Özkan et al. (2023)[18]	Erkenek	9.18	0.48	Geodetic	Block model
Özkan et al. (2023)[18]	Pazarcık	9.27	0.48	Geodetic	Block model
Yıldız et al. (2020)[19]	Amanos	5.4	2.9	Geodetic	Block model
Yıldız et al. (2020)[19]	Erkenek	7.5	0.7	Geodetic	Block model
Yıldız et al. (2020)[19]	Pazarcık	7.5	1	Geodetic	Block model
Walters et al. (2014)[20]	Erkenek	11	3	Geodetic	2D dislocation
Walters et al. (2014)[20]	Erkenek	7.5	0.1	Geodetic	Block model
Walters et al. (2014)[20]	Pazarcık	11	3	Geodetic	2D dislocation
Walters et al. (2014)[20]	Pazarcık	7.5	0.1	Geodetic	Block model
Aktug et al. (2016)[21]	Amanos	4.5	1.1	Geodetic	Block model
Aktug et al. (2016)[21]	Erkenek	10.5	0.6	Geodetic	Block model
Aktug et al. (2016)[21]	Pazarcık	10.3	0.7	Geodetic	Block model
Cavalié and Jónsson (2014)[22]	Erkenek	13	-	Geodetic	2D dislocation
Cavalié and Jónsson (2014)[22]	Pazarcık	13	-	Geodetic	2D dislocation
Duman & Emre (2013)[7]	Goksun	2.5	-	Geologic	Offsets
Duman & Emre (2013)[7]	Cardak	2.5	-	Geologic	Offsets
Yonlu et al. (2013)[23]	Pazarcık	6.9	1.8	Geologic	Offsets
Meghraoui et al. (2011)[24]	Amanos	5.6	1.7	Geodetic	Block model
Meghraoui et al. (2011)[24]	Erkenek	8.9	0.4	Geodetic	Block model
Meghraoui et al. (2011)[24]	Pazarcık	8.9	0.4	Geodetic	Block model
Herece (2008)[25]	Erkenek	10.1	2.8	Geologic	Offsets
Herece (2008)[25]	Pazarcık	9.2	3.2	Geologic	Offsets
Herece (2008)[25]	Pazarcık	5	1	Geologic	Offsets
Seyrek et al. (2007)[26]	Amanos	2.89	-	Geologic	Offsets

Karabacak (2007)[26]	Amanos	4	-	Geologic	Offsets
Reilinger et al. (2006)[27]	Amanos	6.8	0.4	Geodetic	Block model
Reilinger et al. (2006)[27]	Erkenek	7.8	0.4	Geodetic	Block model
Reilinger et al. (2006)[27]	Pazarcık	7.8	0.4	Geodetic	Block model
Parlak et al. (2004)[28]	Erkenek	4.2	0.9	Geologic	Offsets
Yurtmen et al. (2002)[29]	Amanos	1.3	0.3	Geologic	Offsets
Rojay et al. (2001)[30]	Amanos	4	-	Geologic	Offsets
McClusky et al. (2000)[31]	Erkenek	9	1	Geodetic	Block model
McClusky et al. (2000)[31]	Pazarcık	9	1	Geodetic	Block model
Yürür and Chorowicz (1998)[32]	Erkenek	8.4	0.6	Geodetic	Block model
Yürür and Chorowicz (1998)[32]	Pazarcık	8.4	0.6	Geodetic	Block model
Westaway and Arger (1996)[33]	Pazarcık	5.3	1.4	Geologic	Offsets
Westaway (1994)[34]	Erkenek	13.2	0.7	Geodetic	Block model
Westaway (1994)[34]	Pazarcık	13.2	0.7	Geodetic	Block model
Westaway (1994)[34]	Amanos	7	1	Geodetic	Block model
Saroglu et al. (1992)[7]	Pazarcık	5.4	1.1	Geologic	Offsets

Supplementary References

1. KOERI. Kandilli Observatory and Earthquake Research Institute (KOERI) earthquake catalog. (2023).
2. Ambraseys, N. N. Temporary seismic quiescence: SE Turkey. *Geophysical Journal International* **96**, 311–331 (1989).
3. Balkaya, M., Akyüz, H. S. & Özden, S. Paleoseismology of the Sürgü and Çardak faults - splays of the Eastern Anatolian Fault Zone, Türkiye. *Turkish Journal of Earth Sciences* **32**, 402–420 (2023).
4. Yönlü, Ö. & Karabacak, V. Surface rupture history and 18 kyr long slip rate along the Pazarcık segment of the East Anatolian Fault. *JGS* **181**, jgs2023-056 (2024).
5. Karabacak, V. *et al.* The 2023 Pazarcık (Kahramanmaraş, Türkiye) earthquake (Mw 7.7): implications for surface rupture dynamics along the East Anatolian Fault Zone. *Journal of the Geological Society* **180**, jgs2023-020 (2023).
6. Kondorskaya, N.V. & Ulomov, V.I. Special catalogue of earthquakes of the Northern Eurasia (SECNE). (1999).
7. Duman, T. Y. & Emre, Ö. The East Anatolian Fault: geometry, segmentation and jog characteristics. *Geological Society, London, Special Publications* **372**, 495–529 (2013).
8. Ambraseys, N. N. & Jackson, J. A. Faulting associated with historical and recent earthquakes in the Eastern Mediterranean region. *Geophysical Journal International* **133**, 390–406 (1998).
9. Xu, L. *et al.* The overall-subshear and multi-segment rupture of the 2023 Mw7.8 Kahramanmaraş, Turkey earthquake in millennia supercycle. *Commun Earth Environ* **4**, 1–13 (2023).
10. Palutoğlu, M. & Şaşmaz, A. 29 November 1795 Kahramanmaraş Earthquake, Southern Turkey. *Bulletin of the Mineral Research and Exploration* 10–10 (2017) doi:10.19111/bulletinofmre.314211.
11. Dolan, J. F. *et al.* One tune, many tempos: Faults trade off slip in time and space to accommodate relative plate motions. *Earth and Planetary Science Letters* **625**, 118484 (2024).
12. Gauriau, J. & Dolan, J. Comparison of geodetic slip-deficit and geologic fault slip rates reveals that variability of elastic strain accumulation and release rates on strike-slip faults is controlled by the relative structural complexity of plate-boundary fault systems. *Seismica* **3**, (2024).
13. Ambraseys, N. N. Value of Historical Records of Earthquakes. *Nature* **232**, 375–379 (1971).
14. Barbot, S. *et al.* Slip distribution of the February 6, 2023 Mw 7.8 and Mw 7.6, Kahramanmaraş, Turkey earthquake sequence in the East Anatolian Fault Zone. *Seismica* **2**, (2023).
15. Goldberg, D. E. *et al.* Rapid Characterization of the February 2023 Kahramanmaraş, Türkiye, Earthquake Sequence. *The Seismic Record* **3**, 156–167 (2023).
16. Jia, Z. *et al.* The complex dynamics of the 2023 Kahramanmaraş, Turkey, Mw 7.8-7.7 earthquake doublet. *Science* **381**, 985–990 (2023).

17. Li, S. *et al.* Source Model of the 2023 Turkey Earthquake Sequence Imaged by Sentinel-1 and GPS Measurements: Implications for Heterogeneous Fault Behavior along the East Anatolian Fault Zone. *Remote Sensing* **15**, 2618 (2023).
18. Özkan, A., Yavaşoğlu, H. H. & Masson, F. Present-day strain accumulations and fault kinematics at the Hatay Triple Junction using new geodetic constraints. *Tectonophysics* **854**, 229819 (2023).
19. Serhan Yıldız, S. *et al.* Determination of recent tectonic deformations in the vicinity of Adana–Osmaniye–Hatay–Gaziantep triple junction region by half-space modeling. *Comptes Rendus. Géoscience* **352**, 225–234 (2020).
20. Walters, R. J., Parsons, B. & Wright, T. J. Constraining crustal velocity fields with InSAR for Eastern Turkey: Limits to the block-like behavior of Eastern Anatolia. *Journal of Geophysical Research: Solid Earth* **119**, 5215–5234 (2014).
21. Aktug, B. *et al.* Slip rates and seismic potential on the East Anatolian Fault System using an improved GPS velocity field. *Journal of Geodynamics* **94–95**, 1–12 (2016).
22. Cavalié, O. & Jónsson, S. Block-like plate movements in eastern Anatolia observed by InSAR. *Geophysical Research Letters* **41**, 26–31 (2014).
23. Yönlü, Ö., Altunel, E., Karabacak, V. & Akyüz, H. S. Evolution of the Gölbaşı basin and its implications for the long-term offset on the East Anatolian Fault Zone, Turkey. *Journal of Geodynamics* **65**, 272–281 (2013).
24. Meghraoui, M. *et al.* Kinematic modelling at the triple junction between the Anatolian, Arabian, African plates (NW Syria and in SE Turkey). *EGU Geophysical Research Abstracts* **11**, (2013).
25. Herece, E. *Atlas of the East Anatolian Fault*. (2008).
26. Karabacak, V. Quaternary Activity of the Northern Dead Sea Fault Zone. (Eskisehir Osmangazi University, Natural and Applied Sciences Institute, 2007).
27. Reilinger, R. *et al.* GPS constraints on continental deformation in the Africa-Arabia-Eurasia continental collision zone and implications for the dynamics of plate interactions. *J. Geophys. Res.* **111**, B05411 (2006).
28. Parlak, O., Höck, V., Kozlu, H. & Delaloye, M. Oceanic crust generation in an island arc tectonic setting, SE Anatolian orogenic belt (Turkey). *Geological Magazine* **141**, 583–603 (2004).
29. Yurtmen, S., Guillou, H., Westaway, R., Rowbotham, G. & Tatar, O. Rate of strike-slip motion on the Amanos Fault (Karasu Valley, southern Turkey) constrained by K–Ar dating and geochemical analysis of Quaternary basalts. *Tectonophysics* **344**, 207–246 (2002).
30. Rojay, B., Heimann, A. & Toprak, V. Neotectonic and volcanic characteristics of the Karasu fault zone (Anatolia, Turkey): The transition zone between the Dead Sea transform and the East Anatolian fault zone. *Geodinamica Acta* **14**, 197–212 (2001).
31. McClusky, S. *et al.* Global Positioning System constraints on plate kinematics and dynamics in the eastern Mediterranean and Caucasus. *J. Geophys. Res.* **105**, 5695–5719 (2000).

32. Yürür, M. T. & Chorowicz, J. Recent volcanism, tectonics and plate kinematics near the junction of the African, Arabian and Anatolian plates in the eastern Mediterranean. *Journal of Volcanology and Geothermal Research* **85**, 1–15 (1998).
33. Westaway, R. O. B. & Arger, J. A. N. The Gölbaºi basin, southeastern Turkey: a complex discontinuity in a major strike-slip fault zone. *JGS* **153**, 729–744 (1996).
34. Westaway, R. Present-day kinematics of the Middle East and eastern Mediterranean. *Journal of Geophysical Research: Solid Earth* **99**, 12071–12090 (1994).
